# Acute Cycling Exercise Induces Changes in Red Blood Cell Deformability and Membrane Lipid Remodeling

**DOI:** 10.3390/ijms22020896

**Published:** 2021-01-18

**Authors:** Travis Nemkov, Sarah C. Skinner, Elie Nader, Davide Stefanoni, Mélanie Robert, Francesca Cendali, Emeric Stauffer, Agnes Cibiel, Camille Boisson, Philippe Connes, Angelo D’Alessandro

**Affiliations:** 1 Department of Medicine, University of Colorado Anschutz Medical Campus, Aurora, CO 80045, USA; travis.nemkov@cuanschutz.edu (T.N.); davide.stefanoni@cuanschutz.edu (D.S.); francesca.cendali@cuanschutz.edu (F.C.); 2 UNC Blood Center, University of North Carolina, Chapel Hill, NC 27599, USA; scs9yh@virginia.edu; 3 Inter-University Laboratory of Biology of Motor Function EA7424, Vascular Biology and the Red Blood Cell Team, Claude Bernard University Lyon 1, University de Lyon 1, 69100 Villeurbanne, France; elie.nader@free.fr (E.N.); melanie.robert@erytech.com (M.R.); emeric.stauffer@chu-lyon.fr (E.S.); camille.boisson2@gmail.com (C.B.); 4 Laboratory of Excellence (Labex GR-Ex), PRES Sorbonne, 75015 Paris, France; 5 Erytech Pharma, 69008 Lyon, France; agnes.cibiel@erytech.com; 6 Department of Functional Respiratory Testing, Croix-Rousse Hospital, Hospices Civils of Lyon, 69002 Lyon, France; 7 Institute of Universities of France (IUF), 75015 Paris, France

**Keywords:** red blood cell, deformability, cycling, exercise, metabolomics, lipidomics

## Abstract

Here we describe the effects of a controlled, 30 min, high-intensity cycling test on blood rheology and the metabolic profiles of red blood cells (RBCs) and plasma from well-trained males. RBCs demonstrated decreased deformability and trended toward increased generation of microparticles after the test. Meanwhile, metabolomics and lipidomics highlighted oxidative stress and activation of membrane lipid remodeling mechanisms in order to cope with altered properties of circulation resulting from physical exertion during the cycling test. Of note, intermediates from coenzyme A (CoA) synthesis for conjugation to fatty acyl chains, in parallel with reversible conversion of carnitine and acylcarnitines, emerged as metabolites that significantly correlate with RBC deformability and the generation of microparticles during exercise. Taken together, we propose that RBC membrane remodeling and repair plays an active role in the physiologic response to exercise by altering RBC properties.

## 1. Introduction

Maintaining adequate blood flow and tissue perfusion is crucial during exercise because macronutrients, oxygen, carbon dioxide, and metabolic waste products are all transported in the blood. Owing to increased muscle oxygen demand during exercise, exercise-induced physiological changes (e.g., increased heart rate) may increase the mechanical and shear stresses experienced by red blood cells (RBCs) in the bloodstream; such stress may modulate blood rheology and RBC properties, which could in turn impact on blood flow and tissue perfusion. For example, RBCs must be highly deformable to pass through the smallest capillaries and deliver oxygen to the tissues [1]. Moreover, decreased RBC deformability and increased RBC aggregation can cause blood viscosity to increase, thereby altering blood flow and impacting exercise performance [2,3]. Previous studies have shown that acute cycling exercise decreases RBC deformability [4,5,6,7,8,9,10,11]. One cause of this change could be directly or indirectly related to lactate accumulation. Evidence suggests that increased lactate ion concentration and decreased pH could lead to RBC dehydration and activate cationic channels in the RBC membrane, resulting in RBC shrinkage, thereby decreasing RBC deformability [12,13,14,15]. In addition, findings from several studies suggest that oxidative stress could also contribute to reductions in RBC deformability via membrane lipid and protein oxidation [2,16].

Oxidative stress is also a recognized trigger of eryptosis or suicidal erythrocyte death [17]. Eryptosis is characterized by increased intracellular Ca^2+^, cell membrane blebbing, cell shrinkage, and phosphatidylserine (PS) exposure on the RBC membrane surface. These effects have a significant dependence upon metabolic alterations, which are uniquely elicited by different stressors including nutrient depletion, Ca^2+^ overload, and hyperthermia [18]. The onset of eryptosis is associated with the release of microparticles (MPs), membrane-bound vesicles 0.1–1 μm in diameter, which can also be influenced by increased shear stress [19,20,21]. Although shear stress and oxidative stress are both elevated during exercise, previous studies have indicated that short maximal exercise tests do not cause RBC-MPs or markers of eryptosis to increase [22,23]. Similarly, a recent study of endurance-trained athletes showed that a 10 km running trial did not result in elevated levels of RBC-MPs or markers of eryptosis [24]. However, the effect of a prolonged, high-intensity cycling exercise on erythrocyte PS exposure or RBC-MP concentration remains unknown.

As RBCs are devoid of organelles, they are uniquely dependent upon metabolic-associated pathways to cope with oxidative stress. This relationship has been well characterized in RBCs stored in blood banks for transfusion medicine, where accumulating effects of oxidative stress result in cellular damage referred to as the “storage lesion” [25]. In this setting, accumulation of oxidative damage results in decreased deformability and generation of RBC-MPs. A recent study also demonstrated the key role of oxidative stress in RBC deformability and the release of RBC-MPs in the context of sickle cell anemia [26]. The application of omics technologies, including metabolomics, lipidomics, and proteomics, has contributed significantly to the understanding of RBC responses to oxidative stress [27].

Recently, these techniques were also employed to study the complex physiological responses to exercise [28]. These methods have been used to evaluate changes in metabolites related to lipid and carbohydrate metabolism at variable exercise intensities and durations in body fluids including blood, plasma/serum, saliva, and urine [29]. Studies have shown that metabolomics and lipidomics can reveal alterations in oxidative stress [30,31], changes in fuel use during exercise [32,33,34], and distinct metabolic phenotypes corresponding to physiological parameters such as VO_2max_ [35] and lactate clearance capacity [36]. While these studies focused predominantly on metabolic alterations present in plasma, serum, or whole blood, no studies have focused on metabolic alterations in red blood cells (RBCs) during acute exercise specifically or how these cells interact with the circulatory environment of the plasma.

In order to develop a better understanding of the metabolic milieu associated with changes in RBCs during exercise, this study aimed to examine the effects of high-intensity, prolonged exercise on concentrations of metabolites and lipid markers in the plasma and RBC compartments of blood in association with changes in RBC physiology.

## 2. Results

### 2.1. Exercise Testing

The maximal oxygen uptake (VO_2max_) and maximal aerobic power (MAP) values obtained from the graded exercise test indicated that all eight subjects had a high level of aerobic fitness (Table 1). During the 30 min submaximal exercise, subjects pedaled at an average power that corresponded to 71% of MAP. The exercise significantly reduced body weight (−0.88 ± 0.44 kg) and decreased blood oxygen saturation (SpO_2_) (−6.0% ± 3.1%).

### 2.2. Distinct Metabolite Profiles in RBC and Plasma Fractions

**Figure 1:**
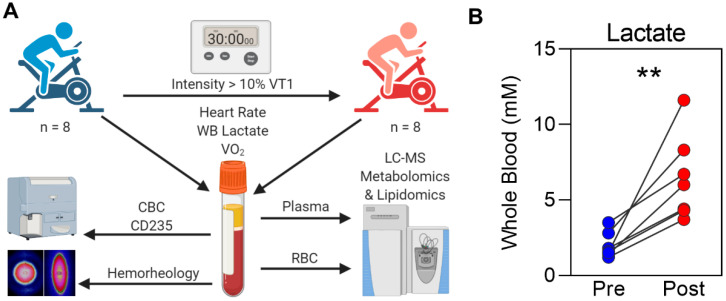
A cycling test to determine responses in metabolism and red blood cell (RBC) properties. (**A**) Eight well-trained male cyclists participated in a 30 min submaximal cycling test (greater than 10% ventilatory threshold 1 (VT1) on an ergometer). In addition to measurement of physiological parameters, whole-blood samples were taken for complete blood counts (CBC) and lactate measurement. Samples were separated into plasma and RBC components. In addition to mass-spectrometry-based lipidomics and metabolomics, RBC samples were also assessed for glycophorin A (CD235) content, deformability, and aggregation properties using ektacytometry. (**B**) Whole-blood (WB) lactate measurements (** *p* < 0.01).

**Figure 2:**
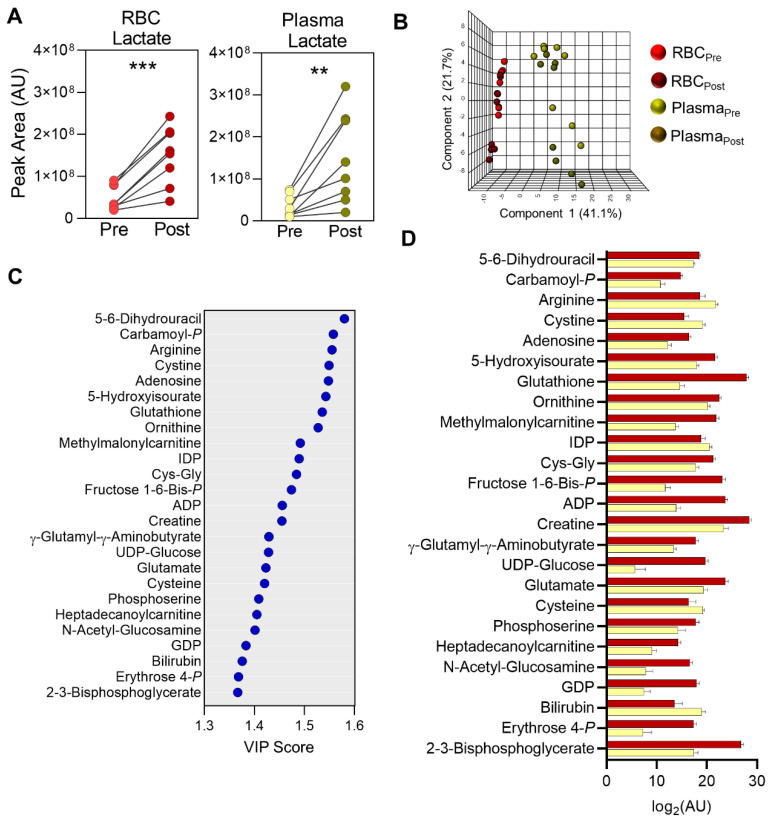
Differential abundance of metabolites in plasma and RBC fractions. (**A**) The abundance of lactate in RBCs and plasma as determined by metabolomics is shown (paired, two-tailed Student’s *t*-test; ** *p* < 0.01, *** *p* < 0.001). (**B**) Partial least-squares discriminant analysis (PLS-DA) of the RBC fraction isolated from cyclists before (Pre) and after (Post) a 30 min cycling test. (**C**) The top 25 metabolites ranked by variable importance in projection (VIP) for Component 1 of the PLS-DA are plotted. (**D**) The peak areas of the top 25 most significantly different metabolites between RBC (red) and plasma (yellow) at the Pre time point are plotted as log_2_(arbitrary units, AUs).

Blood from eight well-trained male athletes was sampled before and 3 min after a 30 min submaximal cycling test (Figure 1A). As expected, whole-blood lactate levels increased in a subject-specific manner during the test (Figure 1B). Lactate is the obligatory byproduct of glycolysis and is produced in part to regenerate nicotinamide adenine dinucleotide (NAD^+^) for the maintenance of glycolytic flux. While lactate measurements during sport performance studies are typically performed using whole blood, this measurement represents the sum of lactate contained predominantly within the plasma and red blood cell (RBC) fractions. In order to measure lactate in the plasma and RBC fractions, as well as the levels of additional metabolites involved in central carbon and nitrogen metabolism, we employed here a high-throughput mass spectrometry-based metabolomics approach. A table of the results is provided in Table S1 (Supplementary Materials). Lactate demonstrated similar concentrations in the plasma and RBC fractions and similar trends in response to the cycling test (Figure 2A). Partial least-squares discriminant analysis (PLS-DA) was employed to classify metabolites that are characteristic of each fraction and to identify compartment-specific responses to exercise (Figure 2B). The metabolic differences between the two matrices of plasma and RBCs contributed to 41.1% of the variance along the Component 1 axis. Meanwhile, the Component 2 axis described 21.7% of the variance pertaining to both inter-subject variability and response to cycling. The top 25 metabolites organized by variable importance in projection (VIP) score for Components 1 and 2 are listed in Figure S1 (Supplementary Materials). The top metabolites that contribute to the Component 1 clustering pattern were ranked by the VIP score (Figure 2C) and plotted by relative abundance (Figure 2D). While amino acids tended to be more abundant in plasma, many charged compounds involved in energy homeostasis including glycolysis (fructose 1,6-bisphoshate, 2,3-bisphosphoglycerate, adenosine diphosphate (ADP)), purine salvage (5-hydroxyisourate, guanosine diphosphate (GDP)), redox homeostasis (glutathione, erythrose-4-phosphate), and glycogen metabolism (uridine diphosphate (UDP)-glucose) were more abundant in the RBC fraction (Figure 2D). A more extensive heatmap of metabolites with statistically significant values (*p* < 0.05) is also provided (Figure S2, Supplementary Materials).

### 2.3. Acute Metabolic Alterations in Plasma during Cycling

**Figure 3:**
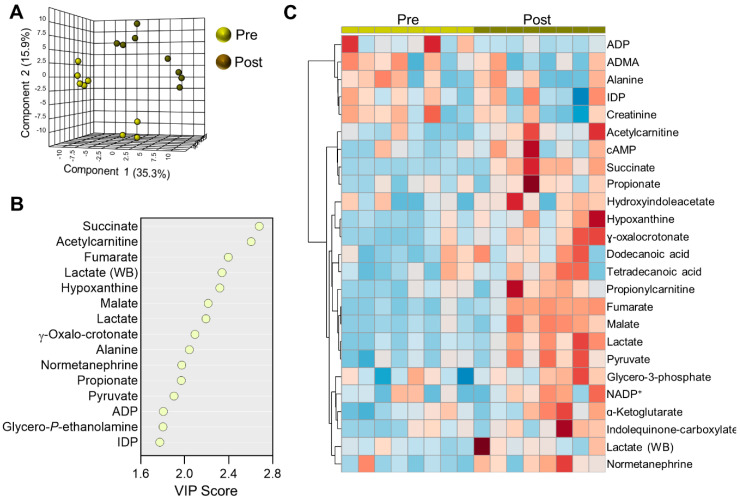
Plasma metabolomics. (**A**) Partial least-squares discriminant analysis (PLS-DA) of the plasma fraction isolated from cyclists before (Pre) and after (Post) a 30 min cycling test. (**B**) The top 15 metabolites ranked by variable importance in projection (VIP) of the PLS-DA are plotted. (**C**) Hierarchical clustering analysis showing the top 25 significant metabolites by *t*-test.

To understand metabolic changes that occur in conjunction with lactate accumulation in each blood compartment, we performed separate multivariate analyses on the respective compartments. PLS-DA of plasma metabolomics data highlighted distinct plasma metabolic profiles before and after cycling, as well as significant inter-subject variability (Figure 3A). The top 25 metabolites organized by VIP score for Components 1 and 2 are listed in Figure S1 (Supplementary Materials). The top 15 metabolites influencing this model largely pertained to energy metabolism including glycolytic metabolites pyruvate and lactate, tricarboxylic acid (TCA) cycle metabolites succinate, fumarate, and malate, and purine salvage metabolites ADP and hypoxanthine (Figure 3B). Despite a substantial degree of difference among cyclists tested in this study, these metabolites followed similar trends upon cycling exercise amongst the entire cohort (Figure 3C). While the levels of alanine tended to be higher at baseline, decreases after cycling in conjunction with increased pyruvate and tricarboxylic acid (TCA) cycle metabolites suggests a rerouting of carbon and nitrogen via transaminase activity in order to meet increased energetic demands. In like fashion, increased levels of dodecanoic and tetradecanoic acid indicate ongoing lipolysis to release free fatty acids, which are conjugated to carnitine for mitochondrial import and oxidation. In support, β-oxidation end-products acetylcarnitine and propionylcarnitine similarly accumulated during cycling. Propionylcarnitine can be formed from the oxidation of branched-chain amino acids and odd-chain fatty acids or directly fueled conjugation of carnitine with propionate. The levels of free propionate also increased during exertion. In addition, the tryptophan catabolites indolequinone-carboxylate and ɣ-oxalocrotonate also increased.

### 2.4. Metabolic Markers of Membrane Remodeling in RBC

**Figure 4:**
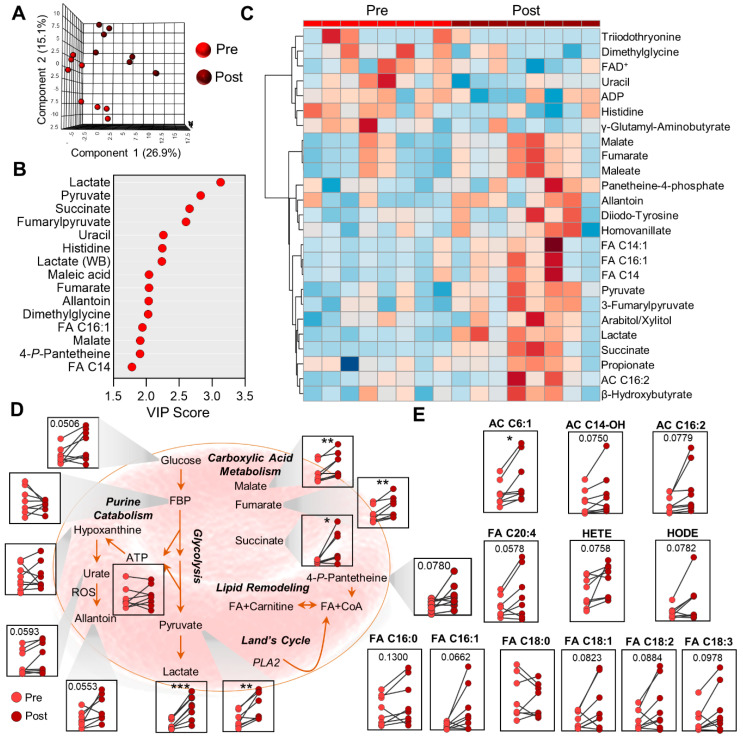
Red blood cell metabolomics. (**A**) Partial least-squares discriminant analysis (PLS-DA) of the RBC fraction isolated from cyclists before (Pre) and after (Post) a 30 min cycling test. (**B**) The top 15 metabolites ranked by variable importance in projection (VIP) of the PLS-DA are plotted. (**C**) Hierarchical clustering analysis showing the top 25 significant metabolites by *t*-test. (**D**) Symbol-and-line plots for glycolysis, purine catabolism, and carboxylic acid metabolism are shown. (**E**) The relative abundances of acylcarnitines (AC) and free fatty acids (FA) are shown. The *y*-axis values are in arbitrary units and not shown. The values from a paired, two-tailed Student’s *t*-test are shown (* *p* < 0.05, ** *p* < 0.01, *** *p* < 0.001).

Multivariate analyses of RBC before and after the cycling test resulted in patterns similar to those of plasma. Specifically, PLS-DA was able to clearly distinguish RBCs before and after cycling in addition to highlighting subgroups within the study cohort, thereby illustrating inter-subject variability in response to exertion (Figure 4A). The top 15 metabolites contributing to this clustering pattern were centrally involved in energy homeostasis (lactate, pyruvate, succinate, fumarate, malate) and fatty acid metabolism (FA C16:1, FA C14, and pantetheine 4-phosphate) (Figure 4B), the relative abundances of which are highlighted in the hierarchical clustering analysis (HCA) of the top 25 significantly changed metabolites by two-tailed Student’s *t*-test (Figure 4C). While this heat map illustrates significant variability within this population, further interrogation of metabolites through network analyses elucidated specific trends regarding energy homeostasis and membrane remodeling. Specifically, elevated levels of glucose, pyruvate, and lactate (Figure 4D) point to enhanced glycolysis in RBCs during exercise presumably to generate ATP for maintenance of ion homeostasis and to subsequently generate (2,3-diphosphoglycerate) DPG [37], which promotes oxygen delivery and the production of which is an oxygen-dependent process [38]. While the analytical platform used here cannot directly measure the levels of DPG (due to co-elution with the 1,3-diphsophglycerate isomer), the steady-state levels of ATP did not significantly change during the 30 min test.

The levels of carboxylic acids such as malate, fumarate, and succinate increased significantly in RBCs during exercise (Figure 4D) and reflect increased levels in the plasma (Figure 3C). These compounds can be transported into RBCs from their circulating environment via carboxylic acid transporters [39], and they are involved in maintaining NAD^+^/NADH ratios in RBCs [40]. However, it is also worth noting that cytosolic isoforms of TCA cycle enzymes are present and active in mature RBCs as a function of oxygen saturation [40].

In addition to glycolytic and carboxylate metabolites, we observed elevations in the levels of many free fatty acids and acylcarnitines (FA and AC, respectively, Figure 4E) to varying degrees in most subjects after cycling. In conjunction with subtle increases in the coenzyme A (CoA) precursor pantetheine 4-phosphate (Figure 4D), these results indicate the activation of membrane lipid remodeling during this cycling test.

### 2.5. Formation of Red Blood Cell Microparticles during Exercise

**Figure 5:**
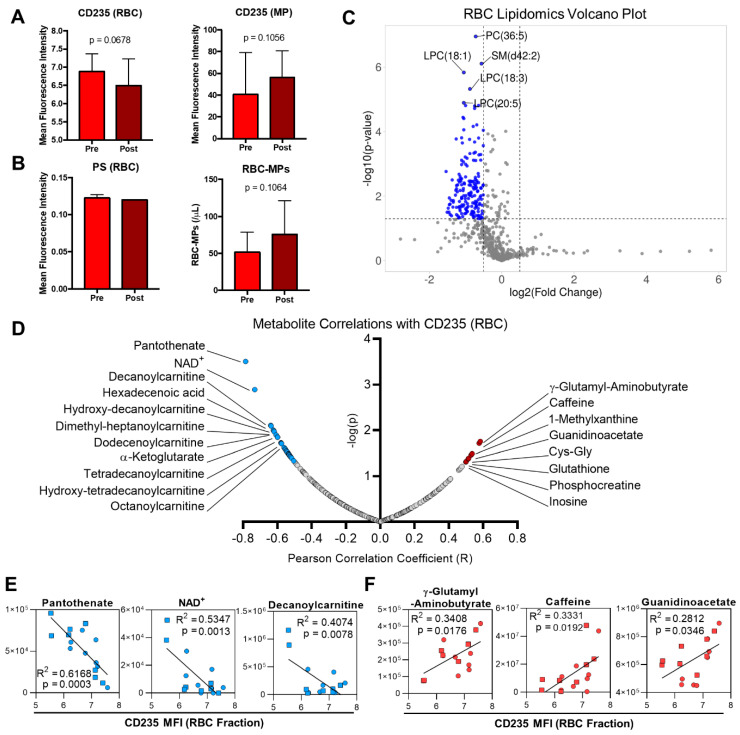
Red blood cell microparticle formation and associated lipid and metabolite features. (**A**) CD235 in the RBC fraction (left) and microparticle (MP) fraction (right), measured in mean fluorescence intensity (MFI) by flow cytometry. (**B**) Phosphatidylserine (PS) exposure on RBCs (left) and total RBC-MPs, quantified by co-labeling PS and glycophorin A (CD235a). (**C**) A volcano plot of global lipidomic results for RBCs is shown. Fold change was calculated as the ratio of Post/Pre values. (**D**) The top Pearson correlates with CD235 MFI in the RBC fraction. (**E**) The top three negative correlates with RBC CD235 MFI are plotted. (**F**) The top three positive correlates with RBC CD235 MFI are plotted. Values from the Pre (○) and Post (□) time points are indicated. Peak areas for each metabolite are given in arbitrary units (AUs) on the *y*-axis.

As RBCs are anucleate cells and, thus, have a limited capacity to cope with oxidative stress and associated component damage, they can remove damaged proteins by membrane vesiculation [41]. While the glycophorin A (CD235)-associated mean fluorescence intensity (MFI) in the RBC fraction tended to decrease, this measurement tended to increase in the microparticle (MP) fraction, indicating increased vesicle shedding during exercise (Figure 5A). The amount of phosphatidylserine (PS) on the outer RBC membrane, however, remained unchanged (Figure 5B). In line with fraction-specific CD235 measurements, untargeted lipidomics analysis of the RBC fraction revealed multiple lipids that significantly decreased in abundance during the cycling test (Figure 5C). Pearson correlation analysis with CD235 content in the RBC fraction highlighted multiple metabolites with significant (*p* ≤ 0.05) associations (Figure 5D). Metabolites with the highest significant negative correlations include free fatty acids, acylcarnitines, NAD^+^, and the CoA precursor pantothenate, with representative correlation plots shown for the top three negative correlates (Figure 5E). Meanwhile, oxidative stress-associated metabolites such as γ-glutamyl-aminobutyrate, Cys–Gly, glutathione, and inosine were amongst the top positive correlates, and representative plots for the top three positive correlates are shown (Figure 5F).

### 2.6. Increased White Blood Cells (WBCs), RBCs, Hemoglobin (Hb), and Hematocrit (HCT) Following Exercise

WBC, RBC, hemoglobin, and hematocrit increased following exercise, while no significant changes were observed in platelets, mean corpuscular volume (MCV), mean corpuscular hemoglobin concentration (MCHC), or red blood cell distribution width (RDW) (Table 2). Increased hematocrit may be in part explained by sudation-driven water loss, as the sum of the total metabolite peak areas for each subject increased after exercise, while the total body weight decreased during the 30 min test (Figure S3, Supplementary Materials). In addition to dehydration, increased catecholamines during exercise cause a recruitment of WBCs into the circulation [42], with a correlation between WBC increase and heart rate after a short period of intense cycling [43]. Furthermore, exercise also results in increased release of platelets into circulation from the liver, lungs, and spleen [44].

### 2.7. Reduced RBC Deformability and Increased RBC Aggregation during Exercise

**Figure 6:**
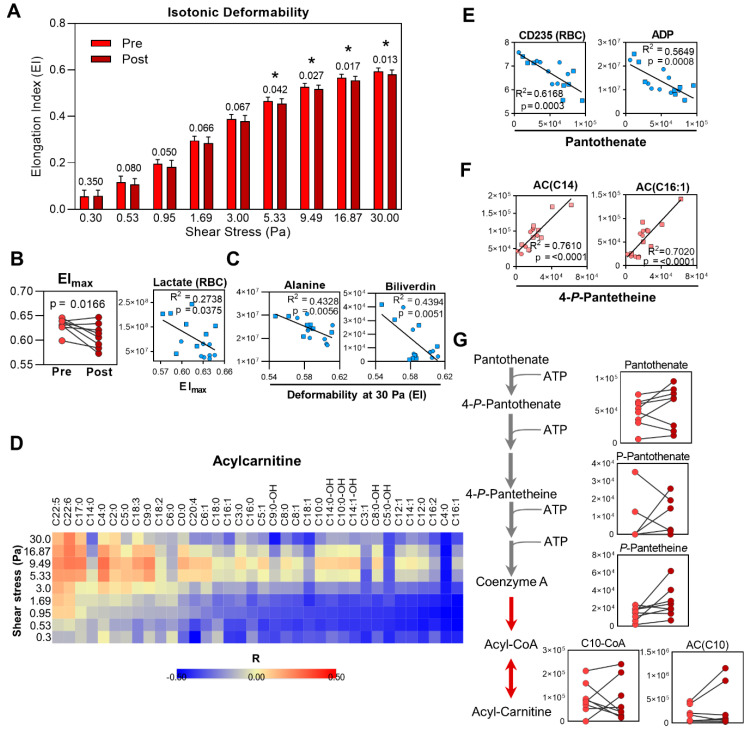
Red blood cell deformability changes during exercise. (**A**) RBC deformability over a gradient of shear stress pressures using samples isolated before (Pre) and after (Post) cycling is shown, with corresponding *p*-values indicated above the bars at each shear stress pressure. Statistically significant differences (*, *p* < 0.05) are indicated. (**B**) Maximum elongation index (EI_max_) at Pre and Post time points is shown (left). The Pearson correlation plot between RBC lactate (*y*-axis given in arbitrary units, AUs, for lactate) and EI_max_ at the Pre (○) and Post (□) time points is shown. (**C**) Correlation plots for alanine and biliverdin with deformability at 30 Pa. The measured peak areas (AUs) for each metabolite are indicated on the respective *y*-axis. (**D**) A heat map showing the Pearson correlation coefficients between various Acylcarnitines measured by metabolomics and deformability over the shear stress gradient is shown. Values are colored from blue to yellow to red according the correlation coefficient (*R*) of −0.5 to 0 to 0.5, respectively. Correlation plots are shown for the top two correlating parameters with (**E**) pantothenate and (**F**) 4-phospho-panteteine. The *x*- and *y*-axis refer to peak areas (AU) of each respective metabolite. (**G**) A pathway for coenzyme A (CoA) synthesis is shown, along with the corresponding values measured by metabolomics at the Pre (left) and Post (right) time points, given in arbitrary units (AUs) on the *y*-axis.

RBC isotonic deformability decreased after the cycling exercise (Figure 6A) in conjunction with increased RBC aggregation (Table 2). While RBC levels of lactate significantly negatively correlated with maximum RBC deformability, represented as maximum elongation index (EI_max_, *R^2^* = 0.274, *p* = 0.038, Figure 6B), alanine and biliverdin were the two top endogenous correlates with deformability (Figure 6C, Table 3). As a compound class, acylcarnitines broadly correlated with RBC deformability at several shear stress pressures (Figure 6D). Because the acyl chains of these molecules are exchanged between carnitine and CoA during the process of membrane remodeling in RBCs, and given the strong correlations observed between MP formation and the level of the CoA precursor pantothenate (Figure 5E), we then assessed for additional parameters that correlated with metabolites in the CoA synthesis pathway. In addition to the RBC content of CD235 (*R^2^* = 0.6168, *p* = 0.0003), ADP (*R^2^* = 0.5649, *p* = 0.0008) was the second top correlate with pantothenate (Figure 6E). Moreover, the downstream metabolite 4-phospho-pantetheine correlated most strongly with the levels of tetradecanoylcarnitine (*R^2^* = 0.761, *p* < 0.0001) and hexadecenoylcarnitine (*R^2^* = 0.702, *p* < 0.0001) (Figure 6F), suggesting an upregulation of this pathway in association with acylcarnitine content (Figure 6G).

## 3. Discussion

Given the central role RBCs play in gas exchange, studies into the effects of exercise on RBC abundance (either decreased due to hemolysis and eryptosis or increased due to activated erythropoiesis), morphological characteristics, and hemorheological parameters (including deformability and aggregation) have been ongoing over the past few decades [45]. Intracellular RBC metabolism, however, has been largely ignored aside from studies on lactate [14,46], ATP [47], and DPG [37,48], all of which are allosteric effectors of hemoglobin (indirectly in the case of lactate) and promote oxygen offloading. While the field of metabolomics has recently contributed progressive findings in sports and exercise science, the majority of these studies have focused on matrices such as plasma, serum, sweat, and urine (reviewed in [28]), thus shifting focus away from exercise-associated effects on RBC metabolism.

We recently reported metabolic responses to short bouts of intense cycling in World Tour competitive cyclists in whole blood using high-throughput mass spectrometry-based metabolomics [36]. While clear signatures were reproduced from other studies including significant accumulations in the blood of tricarboxylic acid (TCA) cycle metabolites, certain metabolites that emerged as significant correlates to lactate clearance capacity were known intracellular metabolites, including 4’-phosphopantothenate and pantetheine-4’-phosphate. As RBCs make up the majority of circulating cells, we hypothesized that the metabolic status of RBC could be associated with endurance capacity. To expand upon various blood compartment contributions observed in that study, here, we analyzed paired plasma and RBC fractions from eight well-trained male cyclists before and after a 30 min intense bout of cycling. This approach provided supporting evidence of the previous findings and quantified relative abundances of metabolites in paired RBCs and plasma. Furthermore, by assessing metabolic responses in these blood compartments in the context of RBC rheological parameters, we were able to identify metabolic pathways that are associated with RBC deformability and MP generation.

While the increase in RBC lactate published in previous work [46] was also recapitulated here, technical limitations in this study prevented accurate measurement of ATP and DPG, which is traditionally performed using enzymatic assays [49]. However, these techniques, when paired with metabolomics readouts of RBCs, offer insight to both the underlying metabolic changes that occur within RBCs exposed to environmental stimuli such as high-altitude hypoxia and functional measurements of oxygen delivery through P50 measurement and assessment of tissue hypoxia [38,40,50,51,52,53]. These mechanisms are mediated in large part through elevated plasma adenosine signaling, which occurs in response to hypoxic stimuli. In line with these studies, we also observed increase plasma adenosine levels after the 30 min cycling test (Table S1, Supplementary Materials). Future studies should interrogate the effects that these mechanisms may also have on RBC responses during exercise using similar model systems.

Acute high-intensity exercise results in lactate accumulation and oxidative stress. These alterations have been shown to decrease RBC deformability by promoting RBC dehydration and shrinkage, in addition to causing oxidation of membrane lipids and proteins [2,14]. In the present study, we did not observe significant changes in MCV, MCHC, or RDW. These results indicate that the increased lactate accumulation probably did not cause RBC dehydration or the observed decrease in RBC deformability. However, our results showing that RBC deformability decreased following exercise are in accordance with previous studies investigating the effects of cycling efforts on RBC rheology [4,5,6,7,8,9,10,11], thus indicating alternative causes for decreased deformability. While the correlation between lactate and EI_max_ observed in this study was subtle, a stronger and significant correlation was found between the levels of alanine and RBC deformability at all tested shear stresses above 0.95 Pa. This result may indicate the activation of RBC alanine aminotransferase (ALT) to reroute pyruvate toward alanine instead of lactate to cope with its significantly increasing levels during the cycling test. Indeed, this mechanism has been observed in response to excess intracellular accumulation of lactate via the inhibition of monocarboxylate transporters [54], as well as in RBCs during routine blood bank storage [55] or undergoing hemorrhagic shock [39].

Oxidative stress has also been implicated in the process of eryptosis or RBC death [17]. However, previous studies indicated that neither a short maximal exercise [23] nor a 10-km maximal running exercise modified markers of eryptosis [24]. Our results here also showed that PS externalization did not increase following exercise. Therefore, these findings agree with previous studies showing that neither a short maximal exercise test nor an endurance run resulted in increased markers of eryptosis. On the other hand, RBC microparticle generation is considered to be a mechanism that can delay RBC death by removing parts of the membrane containing molecules that serve as a signal for RBC removal [56]. In the current study, CD235-labeled MPs increased following exercise. Therefore, it is possible that, although levels of oxidative stress may have increased during the exercise bout, the release of MPs could explain the lack of PS exposure observed in this study. In support, RBC CD235 levels positively correlated with metabolites involved in oxidative stress management including glutathione, Cys–Gly, and γ-glutamyl-aminobutyrate. In addition, inosine also positively correlated with the amount of CD235 in the RBC fraction. Considering its conversion to hypoxanthine by AMD deaminase 3 (AMPD3) is increased by oxidative stress [53,57] and calcium levels [18,58], these findings support the role of oxidative stress in driving MP formation during acute cycling exercise.

In addition to mitigating the effects of oxidative damage, MP generation changes RBC surface-area-to-volume ratio, which in turn can modulate RBC deformability [59,60]. Increased CD235 in the MP fraction, in conjunction with an overall decrease in RBC lipid levels as observed by lipidomics, indicates that this process can occur during a 30 min period of cycling exercise. Furthermore, the observed changes in free fatty acids, eicosanoids, and acylcarnitines indicated that exercise may cause lipid damage in RBCs, resulting in the need for membrane lipid remodeling. RBCs are incapable of generating phospholipids de novo as these processes occur in the Golgi apparatus and endoplasmic reticulum, which are absent in mature RBCs. However, these cells can repair damaged membrane lipids through the Lands cycle [61]. This process involves removal of damaged acyl chains by a phospolipase and esterification with an undamaged fatty acid by lysophospholipid acyltransferase (LPLAT). Acyl-CoA substrates that fuel acyl chain replacement are generated by long-chain acyl-CoA synthetase, which esterifies CoA to fatty acids taken up by the surrounding environment [61,62]. While this process is ATP-dependent, acyl-CoAs can be converted to acylcarnitines by carnitine palmitoyltransferase (CPT), which are then used as substrates provide undamaged acyl chains to membrane lysophospholipids in an ATP-independent manner [62,63]. While lysophospholipids in particular increase in sickle cell RBCs due to an impaired Lands cycle [64], decreased abundance of lyosphosphatidylcholine (LPC) species after exercise here may indicate either increased membrane lipid repair or removal of damaged lipids by MP generation. This latter explanation is supported by the absence of any lipids that significantly increased during cycling, suggesting an overall decrease in membrane surface area. Therefore, these results suggest either that the stress induced by the exercise regimen in the present study was insufficient to elicit lipid damage or that RBCs may activate membrane remodeling pathways during exercise in conjunction with MP generation to remove damaged cellular components. The consequent decrease in surface-area-to-volume ratio may also contribute to decreased deformability that arises during a single bout of cycling exercise.

Membrane remodeling in RBC depends on the level of CoA and carnitine in the cell. CoA itself is synthesized by pantothenic acid, which can be taken up by RBCs [65] and metabolized to produce CoA [66]. We observed multiple correlations among CoA precursors, MP generation, and RBC deformability. Relative levels of the CoA precursors 4-phosphopantothenate and pantetheine-4-phosphate both increased after cycling exercise in this study. Interestingly, these two metabolites were also able to distinguish professional cyclists with varying degrees of blood lactate accumulation over the same power output gradient [36]. While that study was performed using whole blood, the fact that these metabolites are intracellular and correlate with deformability in this study suggest a possible link between RBC membrane damage and repair mechanisms and cycling endurance capacity. In support, we observed here a relationship between acylcarnitine abundance and RBC deformability. Thus, increased shear stress or oxidative stress experienced by RBC during exercise could induce membrane damage in RBCs that requires activation of remodeling.

While RBCs have an average circulatory lifespan of roughly 120 days [67], the population of circulating cells consists of an age distribution that ranges from cells recently produced through ongoing erythropoiesis to senescent RBCs that contain increasing levels of damage markers and are ultimately removed from circulation [68]. Repeated bouts of exercise could activate repair mechanisms that mitigate some of the damage RBCs accumulate during circulation, centered around management of oxidative stress, as well as repair of damaged lipids and proteins, which would mirror RBC responses to blood bank storage [69,70] and inflammatory diseases such as COVID-19 [71]. Alternatively, exercise may result in a more rapid accumulation of membrane damage and earlier clearance from circulation. While future studies should aim to disentangle the specific mechanisms of how exercise imparts changes to RBCs, heme autooxidation produces reactive oxygen species in the form of superoxide and hydroxyl radicals through ongoing Haber–Weiss and Fenton reactions [25], and it is reasonable to predict that the increased frequency of oxygen on- and offloading associated with exercise-induced elevations in oxygen demand could feasibly produce increased oxidative stress in RBCs. In our study, we observed a positive association between metabolites involved in oxidative stress management and RBC CD235 content, while acylcarnitines and pantothenate were inversely associated with this measurement. These results would suggest that RBCs that experience substantial oxidative stress resort to shedding damaged components in the form of MPs, which alters surface-area-to-volume ratio and, thus, deformability. In support, RBC deformability decreased after a 30 min intense cycling test, pointing to an accumulation of damage during the bout. Indeed, training results in increased RBC turnover [72] and a younger overall RBC population [73]. Furthermore, RBCs from endurance athletes tend to be more deformable [74], which suggests that these cells have more “youthful” characteristics. These results are interesting in that they suggest that senescent RBCs are removed from the bloodstream in response to exercise. While our study does not directly address this question, recent work has indicated that splenic sequestration of RBC with decreased deformability may play an important role in the removal of RBCs that accumulate metabolic impairments during routine storage in the blood bank [75]. In addition, similar responses may serve as a protective mechanism against parasitic (malaria) infection to sequester infected cells marked by their decreased deformability [76]. As such, these results support a model of accelerated RBC aging in vivo caused by persistent exercise, which would result in accelerated removal from circulation of RBCs with limited functional capability. The resultant shorter RBC circulatory lifespans would require enhanced erythropoiesis to generate a population of younger cells that are better equipped to face oxidant challenges, at least in the case of aging in vivo [77,78] and in vitro [79] in a subject age- and gender-dependent fashion [80,81]. Future studies should focus on understanding whether exercise training itself can also generate more robust RBCs that are able to cope with a “harsher” environment. Akin to RBCs produced under hypoxic conditions such as high altitude, which are less capable of managing oxidative stress [82] and the first to be cleared from circulation upon return to normoxia [83], it is possible that persistent exercise also holds the potential to fundamentally change the character of newly produced RBCs.

## 4. Materials and Methods

### 4.1. Study Subjects

Eight well-trained, male cyclists (35 ± 7 years; 65.8 ± 6.4 kg; 176.1 ± 9.0 cm) volunteered to participate in the study after providing informed, written consent. Participants had an average training volume of 6.5 ± 2.2 h/week. Participants had no known cardiovascular, metabolic, or pulmonary diseases. The local ethics committee approved the protocol (Lyon, France, L16-47).

### 4.2. Cycling Exercise

During an initial visit to the lab, each athlete performed an incremental maximal exercise test on a cycle ergometer (CycleOps 400 pro, Madison, WI, USA) in order to determine maximal oxygen consumption (VO_2max_) and maximal aerobic power (MAP). Subjects then returned 1 week later to complete a 30 min submaximal exercise bout on the same cycle ergometer. Venous blood samples were drawn from the antecubital vein at rest and 3 min after the end of the submaximal cycling exercise.

### 4.3. Incremental Maximal Cycling Test

The maximal cycling exercise test began with a 3 min warmup at a power output of 90 W. Immediately following the warmup, the power output was increased by 25 W per minute until the subject reached volitional exhaustion. Heart rate was measured using a polar heart rate monitor (810i, PolarElectro, Kempele, Finland). Oxygen uptake, carbon dioxide production, respiratory exchange ratio, and pulmonary ventilation were measured continuously using a breath-by-breath automated exercise metabolic system (Metamax 3b, Leipzig, Germany). VO_2max_ was calculated as the highest 30 s mean value attained prior to exhaustion. Ventilatory threshold (VT1), which represents the point at which lactate begins to accumulate in the blood causing carbon dioxide production and ventilation to increase, was calculated using the ventilatory equivalents method. This method uses the ventilatory equivalent for oxygen, defined as the liters per minute of air ventilating the lungs divided by the liters per minute of oxygen consumed (VE/VO_2_), and the ventilatory equivalent for carbon dioxide, defined as ventilation (L/min) divided by the liters per minute of carbon dioxide produced (VE/VCO_2_). VT1 is calculated as the intensity of exercise that causes the first rise in VE/VO_2_ without a concurrent rise in the VE/VCO_2_ [84].

### 4.4. Submaximal Cycling Exercise

Subjects returned to the lab on a separate day to complete the submaximal exercise test (Figure 1A). Before the test, subjects completed a 10 min warmup on the cycle ergometer at a self-selected resistance and intensity. The warmup was immediately followed by a 30 min bout of high-intensity exercise at a resistance that was equivalent to 10% above the power output reached at VT1. Polar heart rate monitors were used to monitor heart rate during exercise (810i, PolarElectro, Kempele, Finland). Subjects were provided with 500 mL of water to consume during the test, ad libitum, and the volume of water consumed was measured and recorded. The temperature was kept between 20 and 25 °C, and a fan was positioned directly in front of the subjects (at a distance of approximately 1 m) to help them maintain their body temperature. Subjects were weighed before and after the submaximal cycling exercise to calculate the amount of weight lost during the exercise.

### 4.5. Blood Sampling and Whole Blood Lactate Measurement

Venous blood was sampled before and 3 min after the end of the cycling exercise test. Blood was collected in citrate tubes for eryptosis and microparticle measurements, and ethylenediaminetetraacetic acid (EDTA) tubes for hemorheological and metabolomics measurements. A 5 μL drop of blood was also used immediately to measure lactate concentrations before and after the effort using a Lactate pro device (Prolactate).

### 4.6. Metabolomics and Lipidomics Assessment

#### 4.6.1. Sample Collection

Samples of whole blood, collected in EDTA tubes, were centrifuged for 10 min at 2000× *g* at a temperature of 4 °C. Then, 100 μL aliquots of plasma and red blood cells were frozen at −80 °C.

#### 4.6.2. Sample Preparation

Prior to metabolomics analysis, samples were placed on ice and resuspended with nine volumes of ice cold methanol/acetonitrile/water (5:3:2, *v*/*v*/*v*), and suspensions were vortexed continuously for 30 min at 4 °C. Prior to lipidomics analysis, samples were placed on ice and re-suspended with nine volumes of ice-cold methanol, briefly vortexed, and placed at −80 °C for 30 min. All extracts were isolated from insoluble material by centrifugation at 18,000× *g* for 10 min at 4 °C prior to analysis by UHPLC–MS.

### 4.7. UHPLC–MS Analysis for Metabolomics

Analyses were performed as previously published [85]. Briefly, the analytical platform employs a Vanquish UHPLC system (Thermo Fisher Scientific, San Jose, CA, USA) coupled online to a Q Exactive mass spectrometer (Thermo Fisher Scientific, San Jose, CA, USA). The (semi)polar extracts were resolved over a Kinetex C18 column, 2.1 × 150 mm, 1.7 µm particle size (Phenomenex, Torrance, CA, USA) equipped with a guard column (SecurityGuard^TM^ Ultracartridge–UHPLC C18 for 2.1 mm ID Columns–AJO-8782–Phenomenex, Torrance, CA, USA) using an aqueous phase (A) of water and 0.1% formic acid and a mobile phase (B) of acetonitrile and 0.1% formic acid for positive ion polarity mode, and an aqueous phase (A) of water/acetonitrile (95:5) with 1 mM ammonium acetate and a mobile phase (B) of acetonitrile/water (95:5) with 1 mM ammonium acetate for negative ion polarity mode. The Q Exactive mass spectrometer (Thermo Fisher Scientific, San Jose, CA, USA) was operated independently in positive or negative ion mode, scanning in Full MS mode (2 μscans) from 60 to 900 *m*/*z* at 70,000 resolution, with 4 kV spray voltage, 45 sheath gas, and 15 auxiliary gas. Calibration was performed prior to analysis using the Pierce^TM^ Positive and Negative Ion Calibration Solutions (Thermo Fisher Scientific). Acquired data were then converted from raw to mzXML file format using Mass Matrix (Cleveland, OH, USA). Samples were analyzed in randomized order with a technical mixture injected after every 15 samples to qualify instrument performance. Metabolite assignments, isotopolog distributions, and correction for expected natural abundances of deuterium, ^13^C, and ^15^N isotopes were performed using MAVEN (Princeton, NJ, USA). Discovery mode alignment, feature identification, and data filtering was performed using Compound Discoverer 2.0 (Thermo Fisher Scientific).

Graphs, heat maps, and statistical analyses, metabolic pathway analysis, PLS-DA, and hierarchical clustering were performed using the MetaboAnalyst 4.0 package (www.metaboanalyst.com) [86]. Additional graphs were plotted through GraphPad Prism 8 (GraphPad Software Inc., La Jolla, CA, USA).

### 4.8. UHPLC–MS Analysis for Lipidomics

Samples were analyzed as previously published [87]. Briefly, analytes were resolved over an ACQUITY HSS T3 column (2.1 × 150 mm, 1.8 µm particle size (Waters, MA, USA) using an aqueous phase (A) of 25% acetonitrile and 5 mM ammonium acetate and a mobile phase (B) of 90% isopropanol, 10% acetonitrile, and 5 mM ammonium acetate. The column was equilibrated at 30% B, and, upon injection of 10 L of extract, samples were eluted from the column using the following solvent gradient: 0–9 min 30–100% B and 0.325 mL/min, held at 100% B for 3 min at 0.3 mL/min, and then decreased to 30% over 0.5 min at 0.4 mL/min, followed by a re-equilibration hold at 30% B for 2.5 min at 0.4 mL/min. The Q Exactive mass spectrometer (Thermo Fisher Scientific, San Jose, CA, USA) was operated in positive or negative ion mode, scanning in Full MS mode (2 μscans) from 150 to 1500 *m*/*z* at 70,000 resolution, with 4 kV spray voltage, 45 sheath gas, and 15 auxiliary gas. When required, dd-MS2 was performed at 17,500 resolution, automatic gain control (AGC) target = 1 × 10^5^, maximum ion time (IT) = 50 ms, and stepped normalized collision energy (NCE) of 25 and 35 for positive mode, and 20, 24, and 28 for negative mode. Calibration was performed prior to analysis using the Pierce^TM^ Positive and Negative Ion Calibration Solutions (Thermo Fisher). Acquired data were then converted from raw to mzXML file format using Mass Matrix (Cleveland, OH, USA). Samples were analyzed in randomized order with a technical mixture injected incrementally to qualify instrument performance. This technical mixture was also injected three times per polarity mode and analyzed with the parameters above, except collision induced dissociation (CID) fragmentation was included for unknown compound identification. Metabolite assignments were made on the basis of accurate intact mass (sub 5 ppm), isotope distributions, and relative retention times, and they were compared to analytical standards in the SPLASH Lipidomix Mass Spec Standard (Avanti Polar Lipids) using MAVEN (Princeton, NJ, USA). Discovery mode analysis was performed with standard workflows using Compound Discoverer and Lipid Search 4.0 (Thermo Fisher Scientific, San Jose, CA, USA).

### 4.9. Hematological and RBC Rheological Parameters

Hematocrit was measured following blood microcentrifugation using the micro-method. White blood cell (WBC), platelet, and RBC counts, hemoglobin, mean cell volume (MCV), mean corpuscular hemoglobin (MCH), red cell distribution width standard deviation (RDW-SD), and mean corpuscular hemoglobin concentration (MCHC) were measured using a hematological analyzer (XN-350, Sysmex, Japan).

RBC deformability was evaluated at 37 °C over a range of shear stress (0.3–30 Pa), by laser diffraction analysis (ektacytometry), using a laser-assisted optical rotational cell analyzer (LORRCA MaxSis, RR Mechatronics, Hoorn, The Netherlands). This system was previously described in detail (Baskurt 2003 New Guidelines). Briefly, 5 µL of blood was suspended in 1 mL of polyvinylpyrrolidone (PVP; viscosity = 30 cP). The RBC suspension was then sheared using a Couette system, consisting of a rotating transparent outer cylinder and a stationary inner cylinder with a red laser beam. The laser beam is projected from the stationary inner cylinder through the red blood cell suspension, and the resulting diffraction pattern is captured by a charged-coupled device (CCD) video camera and analyzed by computer in order to calculate an elongation index (EI) [88]. An increase in EI reflects improved deformability. Using Lineweaver–Burk modeling, we also determined maximum EI (EI_max_, i.e., EI at infinite shear stress) [89]. RBC aggregation was measured using a Myrenne aggregometer (Myrenne, Roetgen, Germany) with a cone-plate shearing system and integrated infrared light transmission measurement. Blood was sheared at 600 s^−1^ for 10 s to disperse RBC aggregates. Aggregation was then measured during stasis (M) or at a low shear rate of 3 s^−1^ (M1).

### 4.10. Analysis of PS and CD235a Exposure

PS exposure on the outer membrane leaflet of the RBCs was detected using fluorochrome-labeled annexin V, a Ca^2^-dependent phospholipid-binding protein that has a high affinity for PS. RBC membrane glycophorin A (CD235a) level was measured using anti-CD235a antibody. Citrate tubes containing blood collected before and after the submaximal exercise bout were centrifuged (1000× *g*, 5 min at 20 °C), and plasma and buffy coat were removed. The RBC pellets were washed in phosphate buffered saline (PBS) (Corning, 21-040-CVR), and then suspended at 0.2% Hct in the appropriate staining buffer according to the tested conditions. Annexin buffer 1× (Miltenyi, 130-092-820) was used as staining buffer for PS exposure analysis. PBS 1× was used as the buffer for the measurement of CD235a. Depending on the conditions tested, RBC suspensions were incubated with Annexin V–PE (1:11 dilution, Miltenyi, 130-118-363) or anti-CD235a–PE (1:400 dilution, Miltenyi, 130-100-259) for 20 min in the dark at room temperature. Unstained RBCs and isotype controls (1: 51 dilution, REA-PE, Miltenyi) were used as negative controls for PS exposure and CD235a levels, respectively. After incubation, samples were washed twice in their respective staining buffer and analyzed by flow cytometry (Miltenyi, MACSQuant Analyzer 16). A total of 100,000 events, gated for the appropriate forward scatter (FSC), were counted for each sample.

### 4.11. MPs Extraction and Quantification

MPs were quantified as previously reported [90]. Briefly, citrate tubes containing blood collected before and after the bout of submaximal exercise were centrifuged at 1000× *g* for 10 min at 20 °C. Ultracentrifugation (20,000× *g*, 20 min at 20 °C) of the platelet poor plasma was then done to extract the MPs. The supernatant was discarded, and the MP-pellet was washed twice with working buffer (10 mM 4-(2-hydroxyethyl)-1-piperazineethanesulfonic acid (HEPES) pH 7.4, 136 mM NaCl, 5 mM KCl, 2 mM MgCl_2_) that contained 5 mM of EDTA for the first washing step and no EDTA for the second washing step. The suspensions were then stored at −80 °C until the day of analysis.

RBC-MPs were quantified by co-labeling PS and glycophorin A (CD235a). After thawing, MPs were diluted in staining buffer (10 mM HEPES, 3 mM CaCl_2_, pH 7.4). MP suspensions were co-incubated with Annexin V– fluorescein isothiocyanate (FITC) (1:140 dilution, Beckman Coulter, IM3546) and anti-CD235a–PE antibody (1:140 dilution, Miltenyi) for 30 min in the dark at room temperature. After incubation, samples were washed in staining buffer and analyzed by flow cytometry using a MACSQuant Analyzer 16 (Miltenyi Biotec, Bergisch Gladbach, Germany) for MP quantification. The megamix kit was used to standardize MP acquisition gate on the basis of fluorescent microbead size (0.5, 0.9, and 3 μm; Biocytex, Marseille, France) according to the supplier’s instructions. Unstained MPs and single-color-stained tubes were used as controls. RBC-MPs were defined as events that were both smaller than 1 µM and positively labeled with Annexin V–FITC and CD235a–PE.

### 4.12. Statistical Analyses for Hematological, RBC Rheological, PS, CD235a, and MP Data

Statistical tests were conducted using SPSS v.23 (IBM SPSS Statistics, Chicago, IL, USA) with the level of statistical significance set to *p* < 0.05. Paired *t*-tests were used to compare pre- and post-exercise values. Data are expressed as the mean ± standard deviation.

## 5. Conclusions

Here, we described the metabolic and rheological differences in red blood cells (RBC) sampled from well-trained males before and after a controlled, high-intensity 30 min cycling test. RBC demonstrated decreased deformability and increased generation of microparticles after the test. In association with these properties, metabolites involved in oxidative stress response and membrane remodeling and repair emerged as top correlates, thus indicating a metabolic response in RBC to damage resulting from increased circulation and oxygen delivery during exercise. While this study demonstrated only a small degree of statistical significance due to inter-subject variability, future work can expand upon these findings in larger groups. Additionally, understanding the role of biological sex and influence of diet will shed more light onto RBC responses to exercise.

## Figures and Tables

**Table 1:** Subject characteristics.

*n*	8
Age (years)	35 ± 8
Weight (kg)	65.8 ± 6.4
Height (cm)	176.1 ±2.2
VO_2max_ (mL/min/kg)	70.5 ± 5.8
HRmax (BPM)	188 ± 11
MAP (W)	373 ± 39
Percent HRmax at VT1 (%)	85.95 ± 3.4
Percent MAP at VT1 (%)	69.88 ± 5.0
Exercise duration (min)	20.1 ± 1.8

VO_2max_, maximal oxygen uptake; HRmax, heart rate max; MAP, maximal aerobic power; VT1, ventilatory threshold.

**Table 2:** Hematological and hemorheological results.

	Pre	Post
Aggregation M	4.95 ± 2.47	6.03 ± 1.35 *
Aggregation M1	8.35 ± 2.71	9.76 ± 1.30 *
WBCs (10^3^/uL)	4.84 ± 0.93	6.76 ± 1.34 **
RBCs (10^6^/uL)	4.76 ± 0.30	5.01 ± 0.42 *
HGB (g/dL)	14.30 ± 0.90	15.11 ± 1.35 *
HCT %	43.26 ± 2.94	45.56 ± 3.84 *
MCV fL	90.90 ± 3.21	90.97 ± 2.77
MCH pg	30.06 ± 0.96	30.16 ± 0.69
MCHC g/dL	33.07 ± 0.74	33.05 ± 0.86
RDW-SD fL	43.27 ± 2.06	43.26 ± 1.84
PLTs (10^3^/uL)	204.75 ± 43.13	237.25 ± 60.92

WBCs, white blood cells; RBCs, red blood cells, HGB, hemoglobin; HCT, hematocrit; MCV, mean corpuscular volume; MCH, mean corpuscular hemoglobin; MCHC, mean corpuscular hemoglobin concentration; RDW-SD, red cell distribution width standard deviation; PLTs, platelets. * *p* < 0.05, ** *p* < 0.01.

**Table 3:** Metabolite correlates with deformability.

	Alanine	Biliverdin		
Shear Stress	*R^2^*	*p*-Value	*R^2^*	*p*-Value
0.3 Pa	0.1739	0.108	0.2426	0.053
0.53 Pa	0.2507	0.049	0.3691	0.013
0.95 Pa	0.4008	0.009	0.4506	0.004
1.69 Pa	0.3765	0.012	0.3888	0.01
3 Pa	0.3290	0.02	0.3150	0.024
5.33 Pa	0.4162	0.007	0.3115	0.024
9.49 Pa	0.4270	0.006	0.2878	0.032
16.87 Pa	0.4630	0.004	0.3684	0.013
30 Pa	0.4354	0.006	0.4398	0.005

## Data Availability

The data presented in this study are available in Supplementary Materials Table S1.

## Supplementary Materials

Supplementary materials can be found at https://www.mdpi.com/1422-0067/22/2/896/s1: Figure S1. Partial-Least Squares Discriminant Analysis of RBC and Plasma; Figure S2. Hierarchical Clustering Analysis of Plasma and RBC Metabolites; Figure S3. Body Weight and Metabolite Signal as a Function of Cycling Exercise; Table S1. Metabolomics Data.

## Abbreviations

AC	Acylcarnitine
ADP	Adenosine diphosphate
ALT	Alanine aminotransferase
AMPD3	Adenosine deaminase 3
CoA	Coenzyme A
CD235a	Gycophorin A
CPT	Carnitine palmitoyltransferase
DPG	Diphosphoglycerate
EImax	Maximum elongation index
FA	Fatty acid
HCA	Hierarchical clustering analysis
IDP	Inosine diphosphate
LC-MS	Liquid chromatography–mass spectrometry
LPC	Lysophatidylcholine
LPLAT	Lysophospholipid acyltransferase
MAP	Maximal aerobic power
MCHC	Mean corpuscular hemoglobin concentration
MCV	Mean corpuscular volume
MFI	Mean fluorescence intensity
MP	Microparticle
NAD^+^PLS-DA	Nicotinamide adenine dinucleotidePartial least-squares discriminant analysis
PS	Phosphatidylserine
PVP	Polyvinylpyrrolidone
RBC	Red blood cell
RDW	Red blood cell distribution width
SpO2	Blood oxygen saturation
TCA	Tricarboxylic acid
UDP	Uridine diphosphate
VO2max	Maximal oxygen uptake
VIP	Variable importance in project
VT1	Ventilatory threshold 1
WB	Whole blood
WBC	White blood cell
