# Supplementary figures and images for: Acute Cycling Exercise Induces Changes in Red Blood Cell Deformability and Membrane Lipid Remodeling
Source: Int J Mol Sci. 2021 Jan 18;22(2):896. doi: 10.3390/ijms22020896 (PMC7831009; doi:10.3390/ijms22020896)

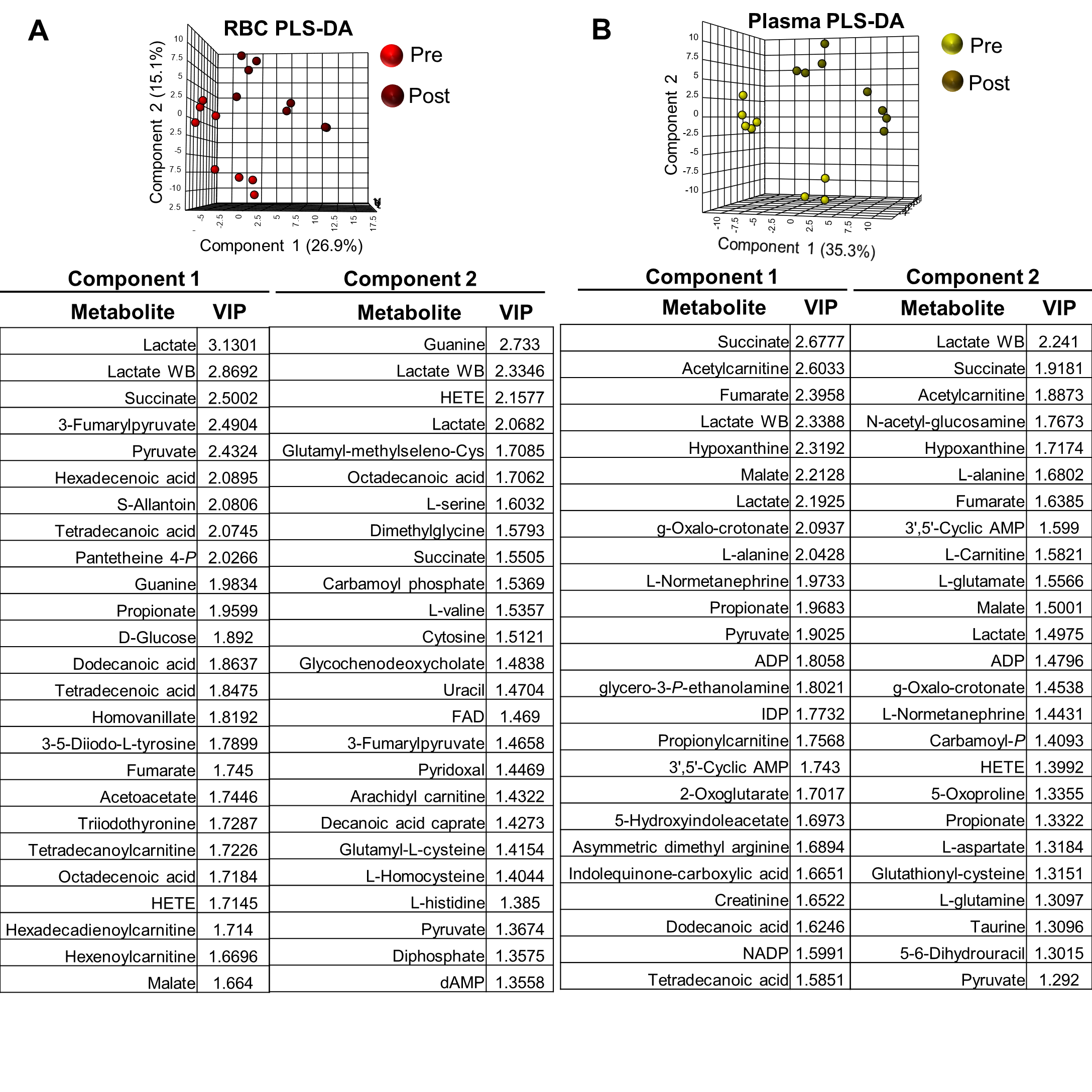

Supplement: Supplementary file 1 [file ijms-22-00896-s001.zip › Supplementary Files/Supplemental Figure 1.tif]

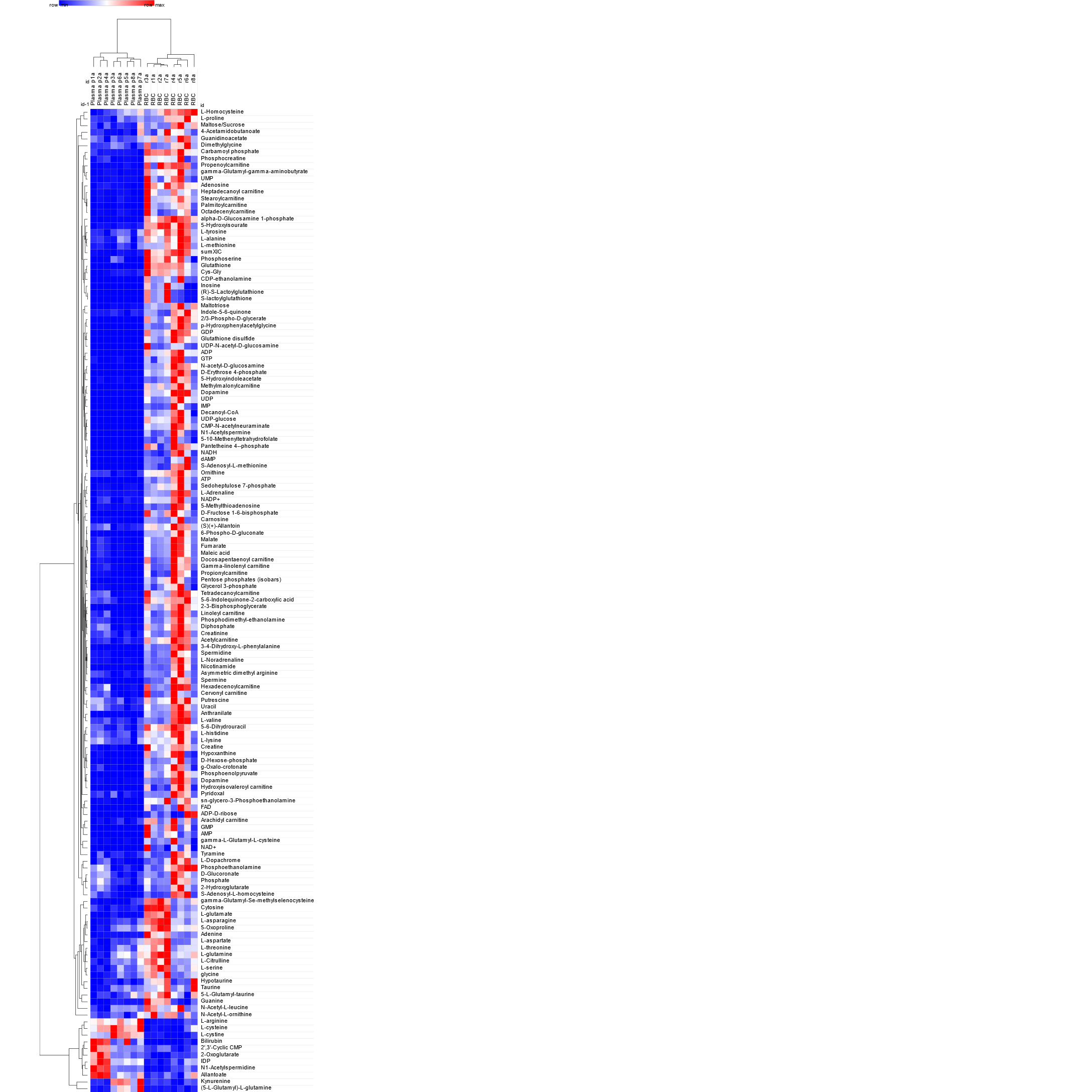

Supplement: Supplementary file 1 [file ijms-22-00896-s001.zip › Supplementary Files/Supplemental Figure 2.tif]

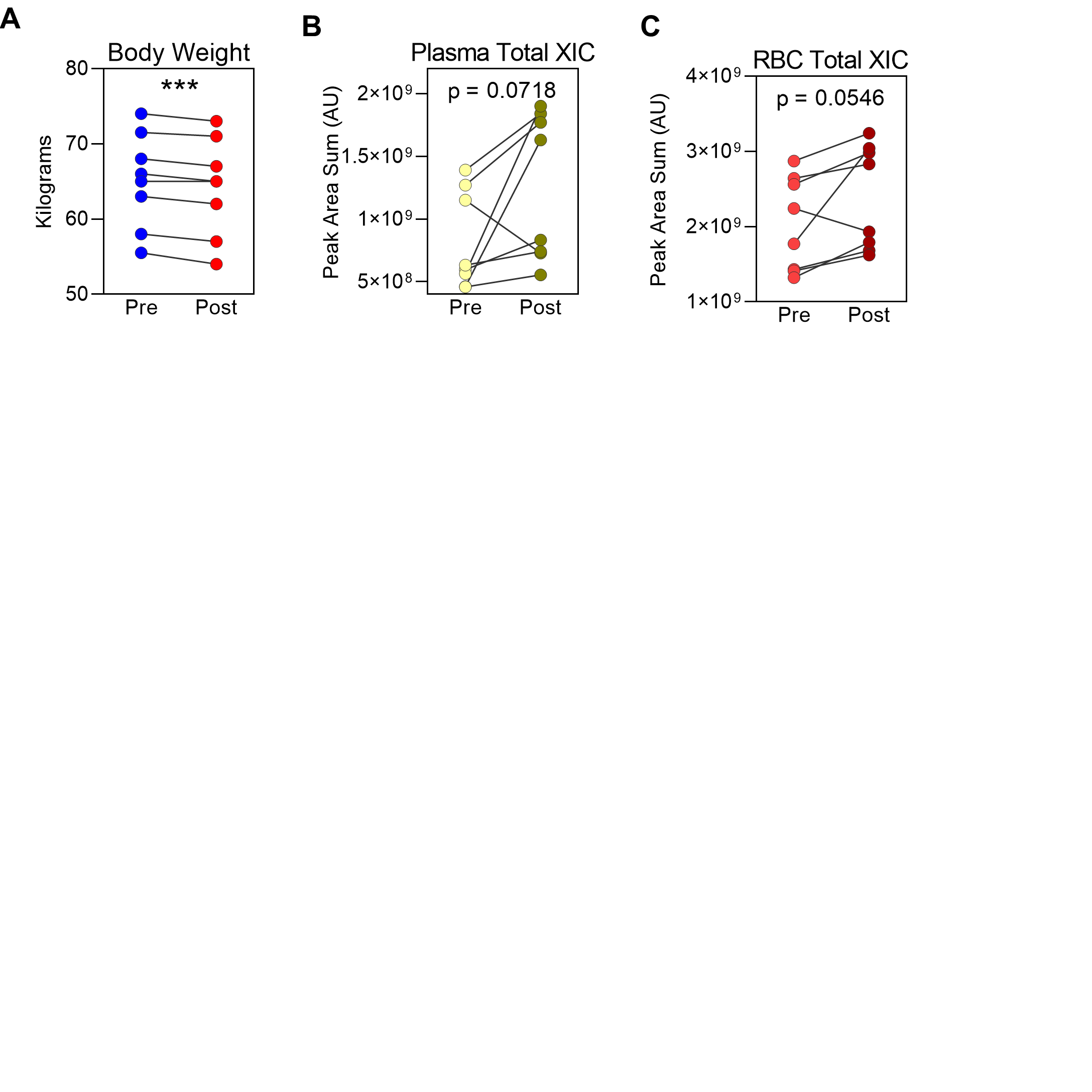

Supplement: Supplementary file 1 [file ijms-22-00896-s001.zip › Supplementary Files/Supplemental Figure 3.tif]
